# Adolescent multiple risk behaviours cluster by number of risks rather than distinct risk profiles in the ALSPAC cohort

**DOI:** 10.1186/s12889-020-8369-6

**Published:** 2020-03-04

**Authors:** Caroline Wright, Jon Heron, Rona Campbell, Matthew Hickman, Ruth R. Kipping

**Affiliations:** Population Health Sciences, Bristol Medical School, Barley House, University of Bristol, Oakfield Grove, Clifton, Bristol, BS8 2BN UK

**Keywords:** ALSPAC, Multiple risk behaviours, Public health intervention, Latent class analysis, Clustering

## Abstract

**Background:**

Experimentation with new behaviours during adolescence is normal. However, engagement in two or more risk behaviours, termed multiple risk behaviours is associated with socioeconomic disadvantage and poor health and social outcomes. Evidence of how adolescents cluster based on their risk behaviours is mixed.

**Methods:**

Latent Class Analysis was used to study patterns of engagement in 10 self-reported risk behaviours (including substance use, self-harm and sexual health) from the Avon Longitudinal Study of Parents and Children (ALSPAC) cohort at ages 15–16 years. Data was available for 6556 adolescents. Associations between risk profile and sex were explored.

**Results:**

A 3-class model for both females and males was deemed to have acceptable fit. Whilst we found evidence of a sex difference in the risk behaviours reported within each class, the sex-specific results were very similar in many respects. For instance, the prevalence of membership of the high-risk class was 8.5% for males and 8.7% for females and both groups had an average of 5.9 behaviours. However, the classes were both statistically dubious, with class separation (entropy) being poor as well as conceptually problematic, because the resulting classes did not provide distinct profiles and varied only by quantity of risk-behaviours.

**Conclusion:**

Clusters of adolescents were not characterised by distinct risk behaviour profiles, and provide no additional insight for intervention strategies. Given this is a more complicated, software-specific method, we conclude that an equally effective, but more readily replicable approach is to use a count of the number of risk behaviours.

## Background

There is growing interest in addressing adolescent multiple risk behaviours (MRB) [1]. MRB is broadly defined as engagement in two or more risk behaviours [2]. Many modifiable MRB (smoking, excessive alcohol intake, poor diet) originate in adolescence, but may become habitual in adulthood, thereby increasing risk of comorbidities and premature mortality. Studies have shown that adolescents involved in one risk behaviour are more likely to be involved in others [3–5]. This can apply both between substances (tobacco, alcohol and illicit drugs) and between substance use and other behaviours such as sexual risk, self-harm and antisocial behaviour [5].

It has been hypothesised that interventions targeting one behaviour are less successful because they do not address co-occurring MRB. Evidence indicates that universal school-based interventions targeting MRB are most efficacious in preventing tobacco smoking, alcohol consumption, illicit drug use, antisocial behaviour and increasing physical activity among young people. Evidence was less conclusive for cannabis use, sexual risk and unhealthy diet [2].

Research considering a wider range of behaviours from multiple domains to inform the scope of interventions is complex, often with strongly related behaviours. Hence data-reduction techniques are adopted, to render the information more manageable. Approaches can be classified as either person-centred or variable-centred. There is often a mathematical equivalence between opposing methods, e.g. Confirmatory Factor Analysis (CFA) and Latent Class Analysis (LCA), the choice of model therefore cannot be motivated solely by data. In public health research, LCA brings the potential to extract and study individuals with differing profiles of behaviour who might respond differently to a targeted intervention [6]. Variable-centred methods include Principle Component Analysis (PCA) and Confirmatory/Exploratory Factor Analysis (CFA/EFA) which simplify observed (co) variation into behavioural traits [7], whilst alternative approaches cluster the behaviours into smaller subsets.

Beginning with variable-centred studies, multiple group CFA was used by de Looze et al. to examine clustering of smoking, drunkenness and cannabis use and early sexual activity among adolescents aged ~ 15 years, across 27 European and North American countries. They found that substance use and early sexual activity loaded on a single underlying cluster consistently across countries [1]. Unfortunately, because they have not considered a wider range of MRB, there may be other co-occurring MRB which we are not aware of. A Dutch study used EFA and CFA to investigate whether a wide range of health and antisocial behaviours clustered. Several separate but interrelated clusters were found. At age 12–15 years one broad cluster and a second cluster comprising alcohol, tobacco smoking and drug use. At age 16–18 years alcohol, unsafe sex, unlawful traffic behaviour and vigorous physical activity; and a second cluster of aggressive behaviour, tobacco smoking, drug use, little sleep and delinquency [8]. It seems that these age groupings have been imposed on the data analysis (rather than them naturally clustering in these groups), so it is difficult to know whether and how age would have impacted the kinds of clusters identified. Hierarchical Agglomerative Cluster Analysis (HACA), was used to explore MRB in Saudi Arabian males, aged ~ 13–19 years. There was evidence for a non-adherence to prevention group (low fruit consumption, less frequent tooth brushing and low physical activity) and a risk behaviour group (high sweets’ consumption, smoking and physical fighting), regardless of age [7]. This analysis is limited by both the small number and type of risk considered as well as the focus on only male adolescents. HACA was also adopted in a study exploring clustering of 17 risk behaviours among Brazilian adolescents aged 13–15 years [9]. This also generated a lack of adherence to preventive behaviours (less frequent hygiene practices, unprotected sex, skipping breakfast, no dental visits), and undertaking risky conduct (current smoking, illegal drug use, no helmet and seatbelt use, high sugar intake, physical fighting and current drinking) and a second unhealthy lifestyle group (sedentary habits, such as insufficient physical activity and eating while watching TV or studying, and diet poor in fruit).

Among the person-centred studies, a New Zealand study used LCA to examine clustering of MRB (alcohol use problems, smoking cigarettes, marijuana use, motor vehicle risk, violence, unsafe sexual health, delinquency, depression and attempted suicide) in a national sample of secondary school students, aged 12–18 years (80% of the sample were aged 14/15 years). The analysis identified a four-class model: the ‘healthy’ group which constituted the majority of students (79.6%), all of whom presented with ≤1 health concerns; the ‘distressed’ group (5.9% of the sample) the majority of whom had depressive symptoms, 48% of whom had attempted suicide in the past year and 52% of whom presented ≥3 health concerns. The ‘risky’ group (10.8% of the sample) with higher rates of risky behaviours, but low rates of emotional concerns. The ‘multiple’ group (3.5% of the sample), reported high levels of both risky behaviours and emotional problems [10]. A similar LCA investigating clustering of MRB among Australians aged ~ 18 years [11], found three classes: moderate risk (52%): moderately likely to binge drink and not eat enough fruit, high probability of insufficient vegetable intake; inactive, non-smokers (24%): high probabilities of not meeting guidelines for physical activity, sitting time and fruit/vegetable consumption, very low probability of smoking; and smokers and binge drinkers (24%): high rates of smoking and binge drinking, poor fruit/vegetable intake. The classes were differentially associated with psychological distress, depression and anxiety. Using LCA, Laxer et al. [12] examined the associations of 15 MRB and overweight/obesity among Canadian adolescents in grades 9 to 12 (age ~ 14–18 years). All groups were more likely to be overweight/obese when compared to the health conscious group: traditional school athletes odds ratio (OR) = 1.15(95%CI:1.03–1.29), inactive screenagers OR = 1.33(95%CI:1.19–1.48) and moderately active substance users OR = 1.27(95%CI:1.14–1.43).

Evidence regarding the clustering of MRB is mixed, with some research finding distinct risk profiles, while others find only broad clusters. This is further obscured when age and sex/gender are considered. Although most studies presented here cover a range of ages across adolescence, two studies de Looze et al. [1] and Champion et al. [11] focus on age 15 and 18 years, respectively, which may be considered limitations to their analysis. While some studies include wide-ranging behaviours, others continue to use only a limited number or type of risk behaviour. Reducing the analysis to similar MRB that are hypothesised to co-occur, runs the risk of missing important relationships that have not been included. It is therefore imperative to test the validity of these profile-specific approaches, incorporating a larger number of divergent risk behaviours for a UK adolescent population.

We aimed to explore the utility of a latent class approach to investigate patterns of MRB using the Avon Longitudinal Study of Parents and Children (ALSPAC) cohort with a view to informing future public health interventions. We chose to examine MRB at age ~ 16 years because adolescent brain development, is second only to infancy as a dynamic period, making it a crucial period of study [13]. In addition, the General Certificate of Secondary Education (GCSE) examinations are completed at age 16 in the UK, determining entrance to post-16 education and university, making it a time of great importance. Further, evidence using ALSPAC data shows that, while not at their highest prevalence, age 16 is when both tobacco and cannabis see their most dramatic increase in use [14]. Similarly, alcohol use is rapidly increasing and antisocial behaviour is at its peak at this age [15].

## Methods

### Participants

Data were drawn from ALSPAC, an ongoing prospective population-based study designed to investigate the effects of a wide range of influences on the health and development of children [16, 17]. Pregnant women resident in Avon, UK with expected dates of delivery 1st April 1991 to 31st December 1992 were invited to take part in the study. The initial number of pregnancies enrolled is 14,541. Of these initial pregnancies, there was a total of 14,676 foetuses, resulting in 14,062 live births and 13,988 children who were alive at 1 year of age. Please note that the study website contains details of all the data that is available through a fully searchable data dictionary and variable search tool (http://www.bristol.ac.uk/alspac/researchers/our-data/). Ethical approval for the study was obtained from the Avon Longitudinal Study of Parents and Children Ethics and Law Committee and local Research Ethics Committees.

### Multiple risk behaviour indicators in adolescence

Data was taken from participants’ responses to both: (i) a previously published self-completed questionnaire issued at a clinic attended at age 15 (median age 15 years and 5 months) and, (ii) a previously published postal questionnaire administered at age 16 (median age 16 years and 7 months) (http://www.bristol.ac.uk/media-library/sites/alspac/documents/questionnaires/CCS-life-of-a-16-plus-teenager.pdf) (see Supplementary Table 1). In previous analyses we have included unprotected sexual intercourse as a thirteenth MRB [18, 19]. However, this was excluded due to its dependency on early sexual behaviour and to avoid structural zeros in the statistical analysis.

### Statistical methods

#### Simple bivariate analysis

Polychoric correlations were derived for each pair of MRB (see Supplementary Table 2). Two behaviours (physical inactivity and TV viewing) were weakly correlated with the other behaviours and each other (≤0.18). We therefore dropped them from our subsequent analysis as they would only diminish model fit.

#### Latent class analyses

The remaining MRB were subjected to a succession of latent class analyses (LCA). LCA assumes that observed associations between variables, are due to a categorical latent variable with two or more classes. Additional classes were added incrementally until the resulting model was deemed acceptable based on a range of statistical and substantive criteria. Analyses were performed separately for females and males because there were some sex differences in the prevalence of MRB (see Supplementary Figure 1).

The Bayesian Information Criterion (BIC) [20] is the most commonly-used fit statistic for comparing LCA models. A function of both the likelihood and number of estimated parameters, the BIC penalises model complexity. Using this statistic, the model with the lowest BIC would be deemed satisfactory.

Conditional independence is an assessment of the remaining association between each pair of measurements once the effect of the latent class variable has been removed. There is currently no accepted threshold for this measure. In addition to a global measure of fit, the individual standardized residuals can be examined to indicate specific areas of poor fit.

The Bootstrap Likelihood Ratio Test (BLRT) and the Lo-Mendell-Rubin (LMR) test statistics [21] both assess change in model fit when adding an additional class. Unlike the LMR, the BLRT makes no distributional assumptions and simulation work has so far shown this measure to be superior [6], however the BLRT can be conservative and may reject all the models considered.

LCA modelling produces a class-assignment probability which describes the confidence each participant can be assigned to each latent class. Entropy, also referred to as classification accuracy, summarises this information as a single measure ranging from zero to one (one indicating no uncertainty). Entropy is of little use in determining the optimal model [22] and can be poor in simulation studies even when the correct model is estimated [23].

Whilst LCA has been promoted as a method to facilitate targeted public health interventions [6] we propose that this is highly dependent on clearly defined, well-separated groups of individuals. Consequently, we regard entropy as an indicator of model *utility* since if it is low and individuals are poorly classified then the resulting classification is of little use as a targeting tool in future interventions.

Analysts often place a limit on the size of the resulting classes. This pragmatic decision is to facilitate any planned future studies or use of LCA. Here we only considered models where all classes contained at least 50 participants.

Both concurrent and face validity are important criteria with which to assess and compare these models. As LCA is an exploratory technique it is important that the within-class profiles are consistent with available evidence. The extraction of implausible classes can be used to justify one model over another. Further, classes which appear similar and cannot be distinguished in relation to any association with key predictors may be of little subtantive use.

#### Missing data

When estimating models with multiple dependent variables, a maximum likelihood (ML) based approach can be employed to address the problem of partial non-response as an alternative to Multiple Imputation (MI), which is based on the missing at random (MAR) assumption i.e. that any differences between the missing and observed values, can be explained by differences in the observed data [24].

LCA permits the inclusion of respondents with ≥1 MRB. To assess the impact of including partially complete data, the same models were estimated on three different subsamples, 1: complete case, 2: all participants with ≤4 missing values and 3: all available data. For the main text of this manuscript we focus on sample 2. Supplementary figures 2 and 3 show comparisons across the three samples.

## Results

### Analysis sample

Ten thousand seven hundred fifty-nine participants were invited to contribute to both a clinic-based (*n* = 9979) and postal questionnaire (*n* = 9510) data collection. Of these, 6556 (61%) (male = 2965/female = 3591) provided some information on MRB. Four thousand eight hundred thirty young people (male = 1981/female = 2849) were missing at most four responses and 2930 (male = 1195/female = 1735) had complete data.

### Latent class models

There was good support for a 3-class model for females and some support for both a 3- and 4-class model for males (model fit statistics are in Supplementary Table 3). Inspection of standardized bivariate residuals for the male and female 3- and 4-class models (Supplementary Figures 4 & 5) showed acceptably low numbers of large residuals for the more parsimonious models. Further, the fourth class was small and made little theoretical sense. Consequently, the 3-class model was chosen for both sexes. We note however that class separation was poor for all models, with entropy typically 0.60–0.70 (entropy > 0.80 is considered good, > 0.90 is considered excellent [25]).

### Within-class profiles of risk behaviour

**Fig. 1:**
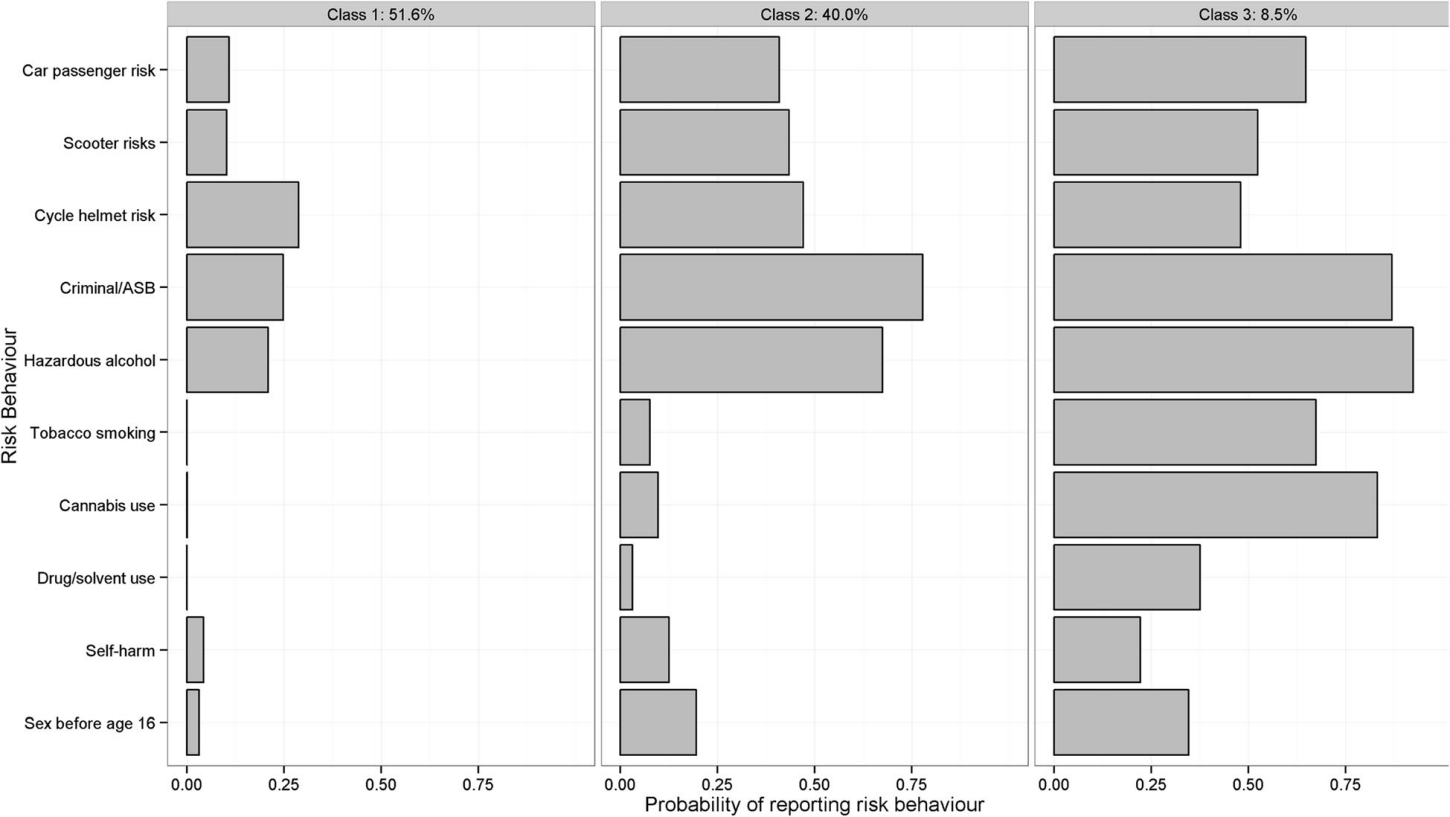
Class specific profiles for three-class solutions for males (sample with up to 4 missing values)

The sex-specific class sizes and average number of MRB reported within each class are shown in Table 1. For males there was a *‘*low risk’ group (51.6%) characterised by few MRB other than a low-to-moderate level of criminality/antisocial behaviour (ASB), alcohol use and lack of helmet wearing, with 1.0 MRB exhibited on average. ‘Medium risk’ (40.0%): characterised by a high probability of criminality/ASB and alcohol use and moderate likelihood of road/injury risks, with 3.3 average MRB. ‘High risk’ (8.5%): similar to medium risk but with the addition of increased chance of tobacco-use and cannabis/other illict drug use, with 5.9 average MRB (see Fig. 1).

**Table 1:** Average number of MRBs reported within each class

	Sample 1: Complete data		Sample 2: ≤ 4 missing values		Sample 3: All available data	
	Prevalence	Average # MRB	Prevalence	Average # MRB	Prevalence	Average # MRB
*Male*	(*n* = 1195)		(*n* = 1981)		(*n* = 2965)	
Class 1 (low risk)	58.3%	1.2	51.6%	1.0	54.2%	1.1
Class 2 (medium risk)	31.8%	3.4	40.0%	3.3	36.3%	3.3
Class 3 (high risk)	9.8%	5.7	8.5%	5.9	9.6%	6.0
*Female*	(*n* = 1735)		(*n* = 2849)		(*n* = 3591)	
Class 1 (low risk)	58.4%	0.9	52.3%	0.8	50.3%	0.8
Class 2 (medium risk)	34.8%	3.1	38.9%	3.0	40.3%	2.9
Class 3 (high risk)	6.8%	6.2	8.7%	5.9	9.3%	6.0

**Fig. 2:**
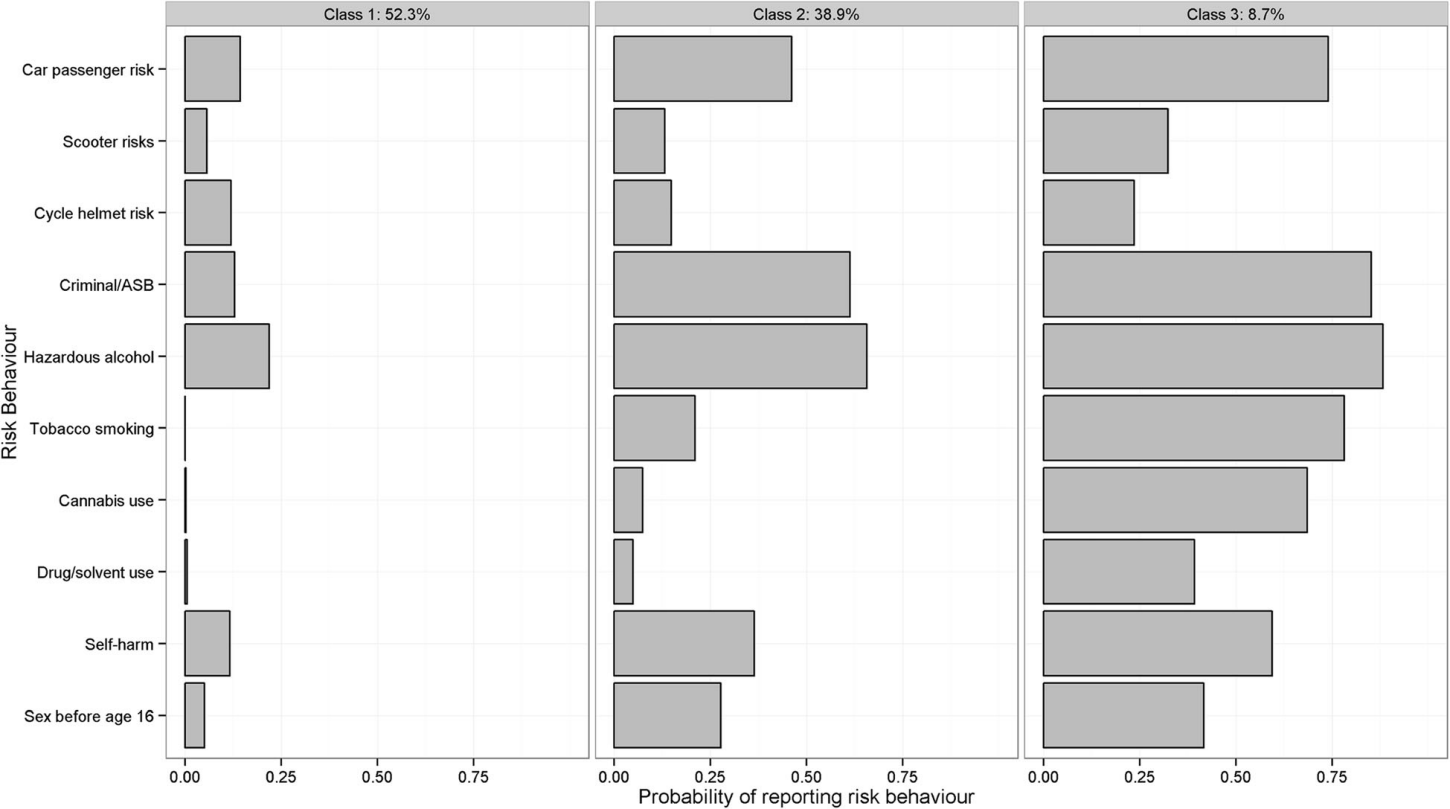
Class specific profiles for three-class solutions for females (sample with up to 4 missing values)

For females there was also a *‘*low risk’ group (52.3%): characterised by few MRB although there were subtle differences with females associated with lower probability of criminality/ASB, scooter risk and helmet risk than males, but a slightly higher probablity of self-harm and sex before age 16 years, with 0.8 MRB exhibited on average. Similarly, the ‘medium risk’group (38.9%) was characterized by moderate-to-large probability of criminality/ASB and alcohol use and moderate likelihood of car-passenger risk but not the other road/injury risks. Self-harm and sex before 16 were also prominent, with 3.0 average MRB. ‘High risk’ (8.7%): all behaviours were raised with the exception of scooter-use and helmet wearing, with 5.9 average MRB (see Fig. 2).

## Discussion

### Main findings

Our LCA found a 3-class model for both females and males and evidence for multiple underlying subgroups. However, classes were not characterised by distinct risk profiles. Rather they varied by quantity of behaviours, with some sex differences in the intensity of these behaviours within classes. The proportion of the cohort in each class and the average number of risk behaviours (~ 1, ~ 3, ~ 6) for each class respectively, was remarkably consistent for females and males. Likewise, the patterning of MRB within each class was broadly similar but with small differences in intensity for scooter risk behaviours, lack of cycle-helmet wearing, criminalty/ASB, self-harm and early-sex (typically more sex-specific behaviours). In addition to indistinct class profiles, class separation (entropy) was poor. Whilst it is possible for the correct model to yield poor class delineation in this way, poor entropy will limit the extent to which such a classification will inform the design of a targeted public health intervention.

We have demonstrated that the utilty of the three classes as an indicator of behaviours to target for MRB prevention, is poor, given the absence of any clustering of types of risk behaviour. The value of the three latent classes in providing empirical support for, or evidence with which to test theories of MRB is also limited.

### Strengths and limitations

This is the first paper that has conducted LCA of a wide range of adolescent MRB in the UK. We used prospectively collected data for a large sample of adolescents, with a wide range of MRB. Further, our choice of behaviours was informed by discussion with two groups of adolescents through the DECIPHer ALPHA young person’s research advisory group (http://decipher.uk.net/public-involvement/young-people/). A limitation is that this research only captures MRB at age 16 years, which may impact the conclusions of the analysis and would benefit from a repeated measures analyses of MRB across adolescence to elicit how engagement in MRB clusters across time.

## Conclusion

Our research calls into question the utility of the clustering approach as a useful way to describe patterns of MRB. The three classes identified were mainly distinguished by the number of MRB engaged in. A better strategy, therefore, is to sum the behaviours to create an overall score. We have shown in a previous analysis that despite individual risk behaviours patterning differently according to sex, females and males engaged in a similar number of MRB [26]. Further, while the associations between individual MRB and socioeconomic status were highly variable, a more consistent relationship was established with MRB score [18]. The evidence points to the volume of behaviours being the critical factor, rather than the types of behaviours engaged in. This has implications for the design of public health interventions aimed at reducing MRB, providing further evidence that MRB co-occur among adolescents, and therefore prevention strategies should focus on multiple rather than single risk behaviours. This is already being encouraged in national policy and while there is some evidence this is being implemented by local authorities among adults [27], more work needs to be done regarding adolescents. Prevention strategies should focus on the quantity, rather than the type of MRB and evidence has shown that interventions targeting multiple-substance use can also be effective for other MRBs, providing an excellent basis for MRB prevention. A recent Cochrane Systematic Review showed that universal school-based interventions are most effective in preventing alcohol consumption, tobacco use, illicit drug use and antisocial behaviour, and increasing physical activity among young people but did not find strong evidence of benefit for family or individual-level interventions for the MRB studied [2]. Therefore, efforts for MRB prevention should focus on developing appropriate school-based interventions.

## Supplementary information

**Additional file 1 **: **Table S1**. Details on the derivation of the risk behaviours. **Table S2**. Polychoric correlations between 12 risk behaviours (males below and females above main diagonal). **Table S3**. Model fit statistics for 10-item Latent Class models across three alternative samples. **Figure S1**. Prevalence of each risk behaviour (complete case sample, *n* = 1195 males / 1735 females). **Figure S2**. Class-specific MRB profiles – Males. **Figure S3**. Class-specific MRB profiles – Females. **Figure S4**. Bivariate residuals for 3 and 4-class males’ models (complete case sample (S1)). **Figure S5**. Bivariate residuals for 3 and 4-class females’ models (complete case sample (S1))

## Data Availability

Please note that the study website contains details of all the data that is available through a fully searchable data dictionary and variable search tool (http://www.bristol.ac.uk/alspac/researchers/our-data/).

## Abbreviations

ALSPAC: Avon Longitudinal Study of Parents and Children
LCA: Latent Class Analysis
MRB: Multiple Risk Behaviour
