# Supplementary material for: Adolescent multiple risk behaviours cluster by number of risks rather than distinct risk profiles in the ALSPAC cohort
Source: BMC Public Health. 2020 Mar 4;20:290. doi: 10.1186/s12889-020-8369-6 (PMC7057689; doi:10.1186/s12889-020-8369-6)
Supplement: Supplementary file 1 — Additional file 1 : Table S1. Details on the derivation of the risk behaviours. Table S2. Polychoric correlations between 12 risk behaviours (males below and females above main diagonal). Table S3. Model fit statistics for 10-item Latent Class models across three alternative samples. Figure S1. Prevalence of each risk behaviour (complete case sample, n = 1195 males / 1735 females). Figure S2. Class-specific MRB profiles – Males. Figure S3. Class-specific MRB profiles – Females. Figure S4. Bivariate residuals for 3 and 4-class males’ models (complete case sample (S1)). Figure S5. Bivariate residuals for 3 and 4-class females’ models (complete case sample (S1)) [file 12889_2020_8369_MOESM1_ESM.docx]

**SUPPLEMENTARY MATERIALS**

**Supplementary Table 1. Details on the derivation of the risk behaviours**

| Health risk behaviour | Definition / how derived |
| --- | --- |
| Physical inactivity | Young person (YP) has typically over the past year exercised < 5 times per week |
| TV viewing | YP spent 3 or more hours watching television on average per day across the week. |
| Car passenger risk | YP had been a car passenger at least once in their lifetime where the driver (a) had consumed alcohol or (b) did not have a valid licence or (c) chose not to wear a seat belt last time travelled in a car, van or taxi |
| Cycle helmet use | If the YP reported that they had last ridden a bicycle within the previous four weeks and they had not worn a helmet on the most recent occasion |
| Scooter risks | YP has driven a scooter off road or with or without a licence (or both) at least once time in lifetime |
| Criminal/Antisocial behaviour | YP self-reported that at least once in the past year they had undertaken at least one of the following:-carried a weapon; physically hurt someone on purpose; stolen something; sold illicit substances to another person; damaged property belonging to someone else either by using graffiti, setting fire to it or deliberately destroying or damaging it in another fashion; subjected someone to verbal or physical racial abuse; or been rude/rowdy in a public place |
| Hazardous alcohol | AUDIT score of 8 or more |
| Tobacco smoking | Regular weekly smoking that is currently smoking at least one cigarette per week. |
| Cannabis use | Occasional cannabis use - “sometimes but less often than once a week” or categories indicating more regular use |
| Drug/solvent use | In the year since their 15^th^ birthday, YP had either been a regular user (i.e. used five or more times) of one or more of amphetamines, ecstasy, LSD, cocaine, ketamine or a variety of inhalants including aerosols, gas, solvents and poppers |
| Self-harm | Young people who said they had purposely hurt themselves in some way in their lifetime. |
| Sexual intercourse prior to age 16 | YP reported having had penetrative sex in the last year and they were under 16 at the time. |

Sources of information:

*15 year clinic*: criminal and antisocial behaviour, sex prior to age 16.

*16 year questionnaire*: physical inactivity, TV viewing, car passenger risk, cycle helmet use, scooter risk, hazardous alcohol drinking.

*Both sources*: weekly tobacco smoking, cannabis use, drug and solvent use, self-harm. For those risk behaviours which were assessed on both occasions the clinic responses (where available) were used for those participants who did not return the questionnaire.

**Supplementary Table 2. Polychoric correlations between 12 risk behaviours (males below and females above main diagonal)**

|  | Physical inactivity | TV viewing | Car passenger risk | Helmet use | Scooter risk | Criminal/ASB | Alcohol | Tobacco | Cannabis | Drugs | Self-harm | Sex prior to age 16 |
| --- | --- | --- | --- | --- | --- | --- | --- | --- | --- | --- | --- | --- |
| Physical inactivity |  | 0.08 | 0.08 | -0.11 | -0.04 | 0.07 | 0.06 | 0.18 | 0.06 | 0.09 | 0.08 | 0.04 |
| TV viewing | 0.05 |  | 0.11 | -0.14 | -0.13 | 0.11 | 0.02 | 0.01 | -0.06 | 0.04 | -0.02 | 0.05 |
| Car passenger risk | 0.00 | 0.01 |  | 0.08 | 0.26 | 0.35 | 0.38 | 0.44 | 0.44 | 0.30 | 0.32 | 0.28 |
| Helmet use | -0.13 | -0.02 | 0.00 |  | 0.14 | 0.01 | 0.00 | 0.05 | 0.19 | 0.23 | 0.00 | -0.04 |
| Scooter risk | -0.08 | 0.03 | 0.30 | 0.31 |  | 0.24 | 0.16 | 0.35 | 0.28 | 0.17 | 0.14 | 0.13 |
| Criminal/ASB | -0.08 | 0.02 | 0.32 | 0.15 | 0.38 |  | 0.42 | 0.56 | 0.54 | 0.46 | 0.40 | 0.46 |
| Alcohol | -0.03 | -0.06 | 0.41 | 0.19 | 0.33 | 0.38 |  | 0.54 | 0.53 | 0.37 | 0.28 | 0.33 |
| Tobacco | 0.14 | 0.12 | 0.43 | 0.14 | 0.33 | 0.49 | 0.51 |  | 0.69 | 0.54 | 0.45 | 0.43 |
| Cannabis | 0.14 | -0.04 | 0.40 | 0.21 | 0.23 | 0.45 | 0.52 | 0.78 |  | 0.71 | 0.42 | 0.32 |
| Drugs | 0.03 | -0.02 | 0.36 | 0.12 | 0.26 | 0.38 | 0.48 | 0.70 | 0.71 |  | 0.45 | 0.22 |
| Self-harm | 0.16 | -0.04 | 0.13 | 0.00 | -0.02 | 0.33 | 0.21 | 0.25 | 0.30 | 0.40 |  | 0.29 |
| Sex prior to age 16 | -0.03 | 0.06 | 0.19 | 0.06 | 0.32 | 0.44 | 0.34 | 0.48 | 0.31 | 0.42 | 0.11 |  |

Complete case sample (n = 1,195 males / 1,735 females).

**Supplementary Table 3. Model fit statistics for 10-item Latent Class models across three alternative samples**

|  |  | Males | | | | | | | Females | | | | | | |
| --- | --- | --- | --- | --- | --- | --- | --- | --- | --- | --- | --- | --- | --- | --- | --- |
| Sample | # class | SSA-BIC | Cond. Indep. | Smallest class | Entropy | -2ΔLL | BLRT p | LMR p | SSA-BIC | Cond. Indep. | Smallest class | Entropy | -2ΔLL | BLRT p | LMR p |
| Complete case  (m:1,195 / f: 1,735) | 1 | 11281.9 | 1861.6 | 1195 | - | - | - | - | 16029.2 | 2762.2 | 1735 | - | - | - | - |
|  | 2 | 10517.2 | 165.2 | 341.8 | 0.718 | 807.6 | <0.001 | <0.001 | 14855.8 | 135.7 | 479.0 | 0.739 | 1220.5 | <0.001 | <0.001 |
|  | 3 | 10442.0 | 57.3 | 117.3 | 0.677 | 118.2 | <0.001 | <0.001 | 14765.6 | 43.8 | 118.5 | 0.692 | 137.3 | <0.001 | <0.001 |
|  | 4 | 10451.7 | 32.5 | 109.3 | 0.743 | 33.4 | 0.037 | 0.016 | 14783.1 | 31.0 | 104.5 | 0.687 | 29.6 | 0.484 | 0.058 |
|  | 5 | 10464.9 | 23.0 | 102.3 | 0.789 | 29.8 | 0.042 | 0.070 | 14801.4 | 23.1 | 34.3 | 0.718 | 28.8 | 0.295 | 0.088 |
|  | 6 | 10483.8 | 14.9 | 55.0 | 0.712 | 24.1 | 0.818 | 0.328 | 14824.8 | 20.3 | 10.7 | 0.743 | 23.8 | † | † |
| Up to 4 missing values  (m:1,981 / f:2,849) | 1 | 17307.0 | 3050.1 | 1981 | - | - | - | - | 24528.0 | 4330.1 | 2849 | - | - | - | - |
|  | 2 | 16169.6 | 246.5 | 593.8 | 0.689 | 1186.0 | <0.001 | <0.001 | 22842.1 | 185.0 | 807.5 | 0.708 | 1738.5 | <0.001 | <0.001 |
|  | 3 | 16039.2 | 94.7 | 167.4 | 0.649 | 179.0 | <0.001 | <0.001 | 22717.5 | 71.4 | 249.4 | 0.628 | 177.1 | <0.001 | <0.001 |
|  | 4 | 16030.5 | 49.8 | 164.1 | 0.625 | 57.3 | 0.139 | <0.001 | 22742.4 | 56.2 | 79.2 | 0.671 | 27.6 | 0.642 | 0.094 |
|  | 5 | 16047.1 | 42.9 | 98.8 | 0.638 | 32.0 | 0.194 | 0.018 | 22766.3 | 37.9 | 74.4 | 0.671 | 28.7 | 0.032 | 0.084 |
|  | 6 | 16067.1 | 20.8 | 93.8 | 0.598 | 28.6 | 0.660 | 0.082 | 22793.1 | 21.7 | 59.8 | 0.694 | 25.7 | † | † |
| Max possible sample  (m:2,965 / f:3,591) | 1 | 22871.0 | 5162.1 | 2965 | - | - | - | - | 29309.2 | 5740.5 | 3591 | - | - | - | - |
|  | 2 | 21157.5 | 334.3 | 776.9 | 0.722 | 1766.4 | <0.001 | <0.001 | 27256.3 | 241.0 | 1000.0 | 0.705 | 2108.0 | <0.001 | <0.001 |
|  | 3 | 20989.9 | 143.0 | 283.6 | 0.628 | 220.6 | <0.001 | <0.001 | 27112.9 | 100.2 | 335.4 | 0.607 | 198.4 | <0.001 | <0.001 |
|  | 4 | 20989.9 | 79.7 | 302.9 | 0.574 | 53.0 | 0.483 | <0.001 | 27137.4 | 82.7 | 158.6 | 0.681 | 30.6 | 0.642 | 0.032 |
|  | 5 | 21007.5 | 53.9 | 220.3 | 0.582 | 35.4 | 0.277 | 0.014 | 27163.4 | 63.6 | 107.4 | 0.695 | 29.1 | 0.033 | 0.066 |
|  | 6 | 21032.4 | 41.7 | 156.4 | 0.579 | 28.1 | 0.397 | 0.100 | 27195.1 | 41.5 | 110.9 | 0.626 | 23.3 | † | † |

†: estimation problems; -2ΔLL= -2*(change in log-likelihood)

SSA-BIC = Sample-size adjusted BIC; BLRT = Bootstrap Likelihood Ratio Test; LMR = Lo-Mendell Rubin test

**Supplementary Figure 1. Prevalence of each risk behaviour (complete case sample, n = 1,195 males / 1,735 females)**

**Supplementary Figure 2. Class-specific MRB profiles - Males**

Sample 1: Complete case (n = 1.195)


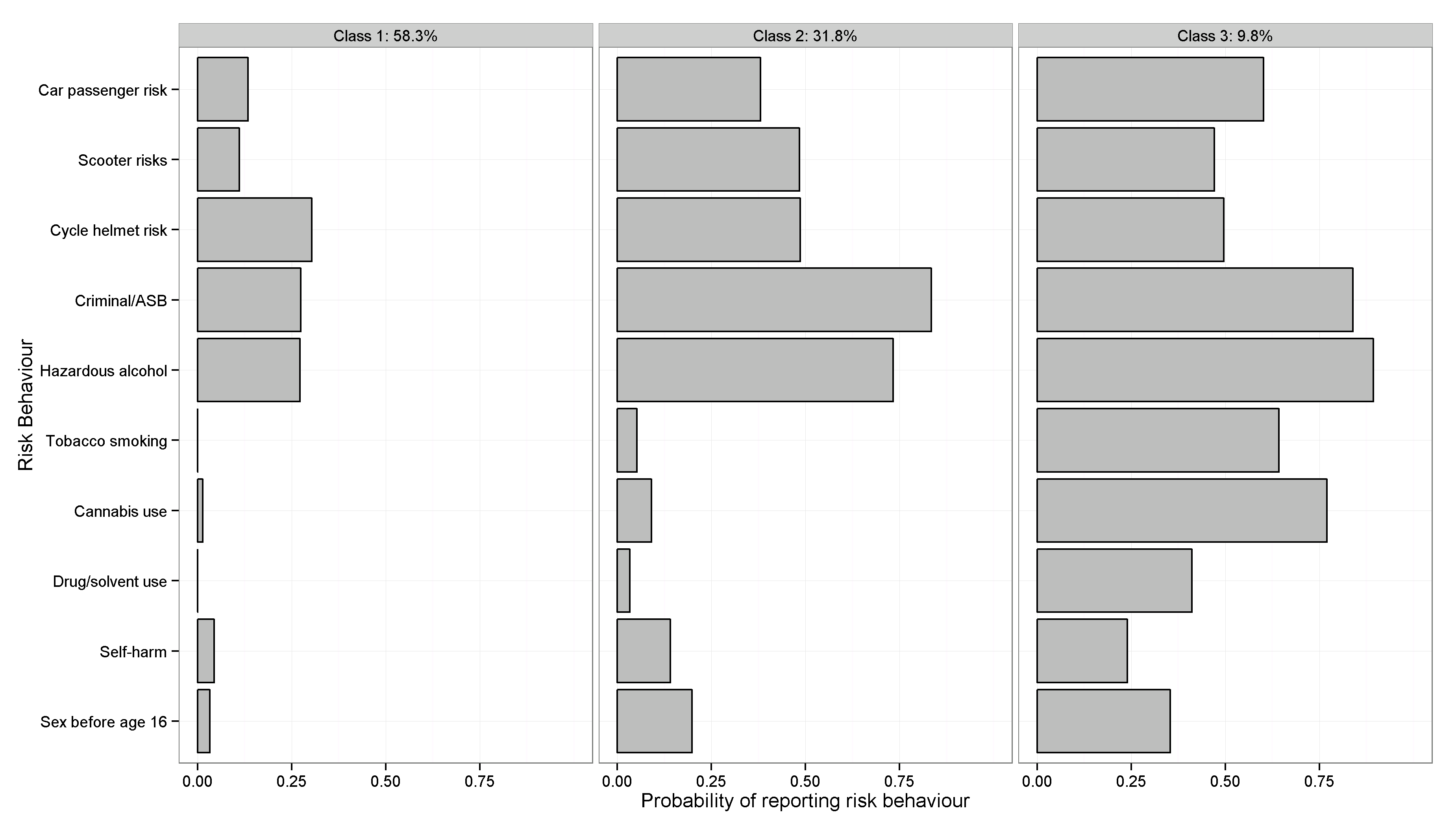


Sample 2: Up to 4 missing (n = 1,981)


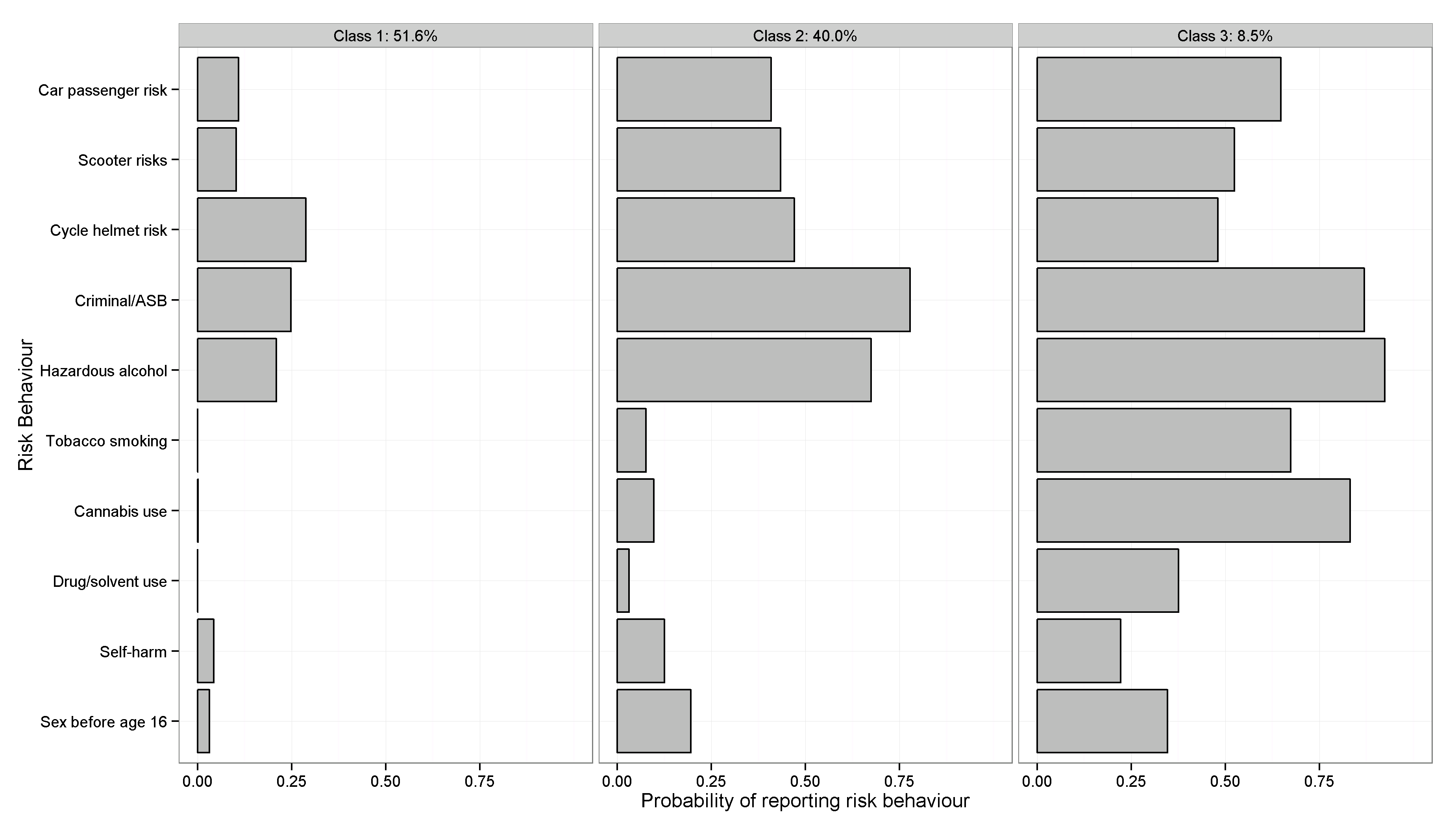


Sample 3: All available data (n = 2,965)


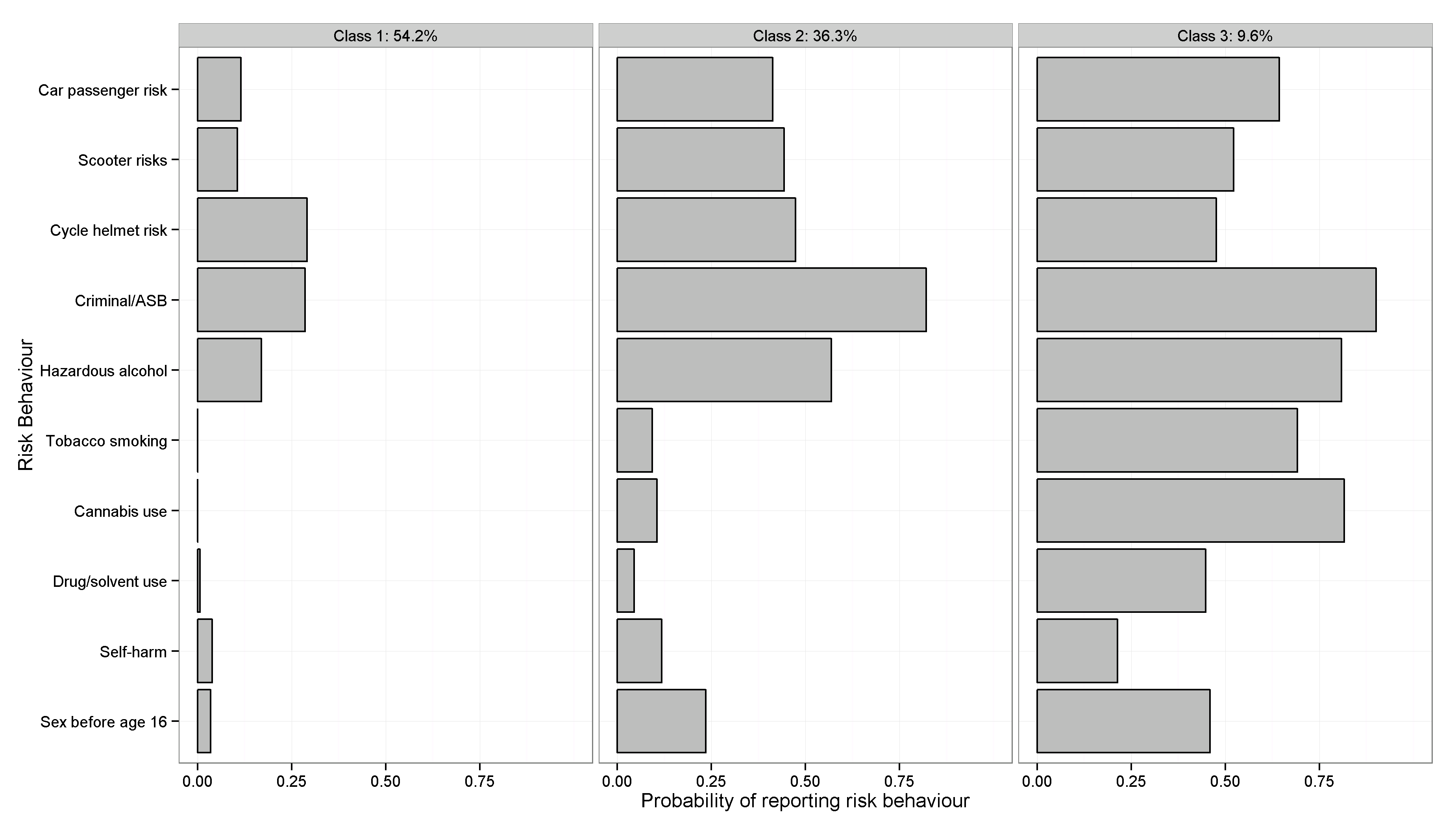


**Supplementary Figure 3. Class-specific MRB profiles - Females**

Sample 1: Complete case (n = 1,735)


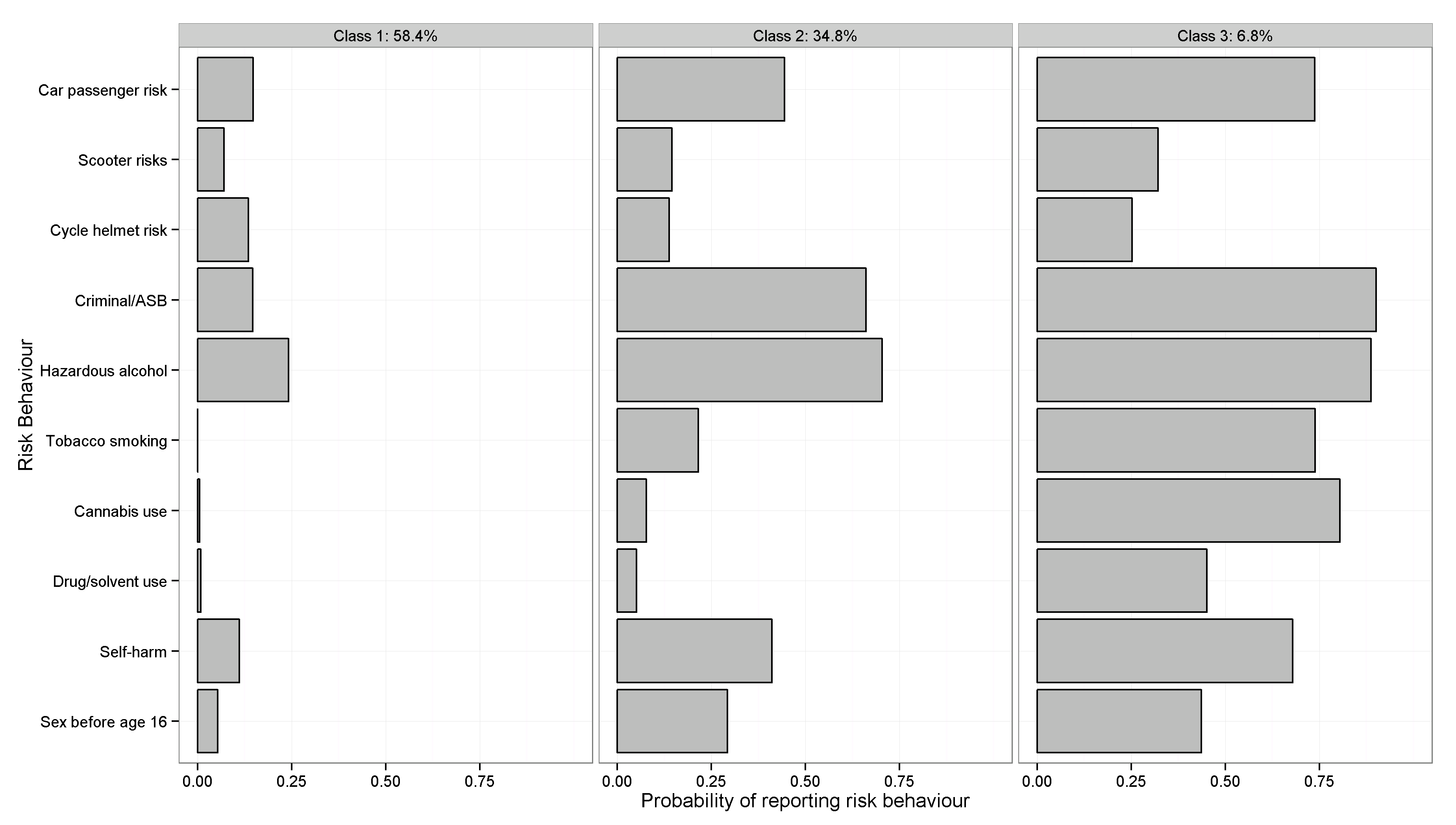


Sample 2: Up to 4 missing (n = 2,849)


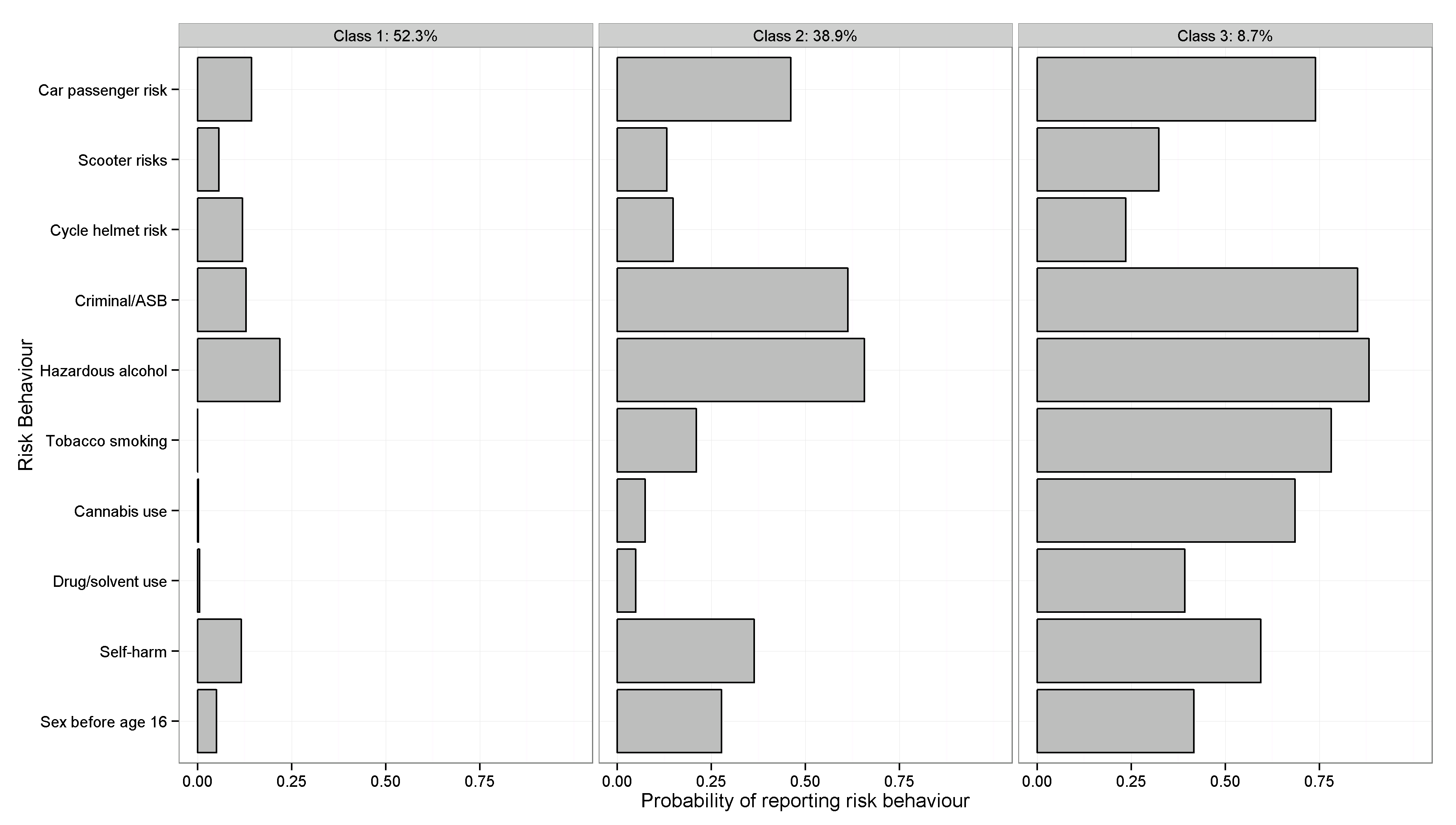


Sample 3: All available data (n = 3,591)


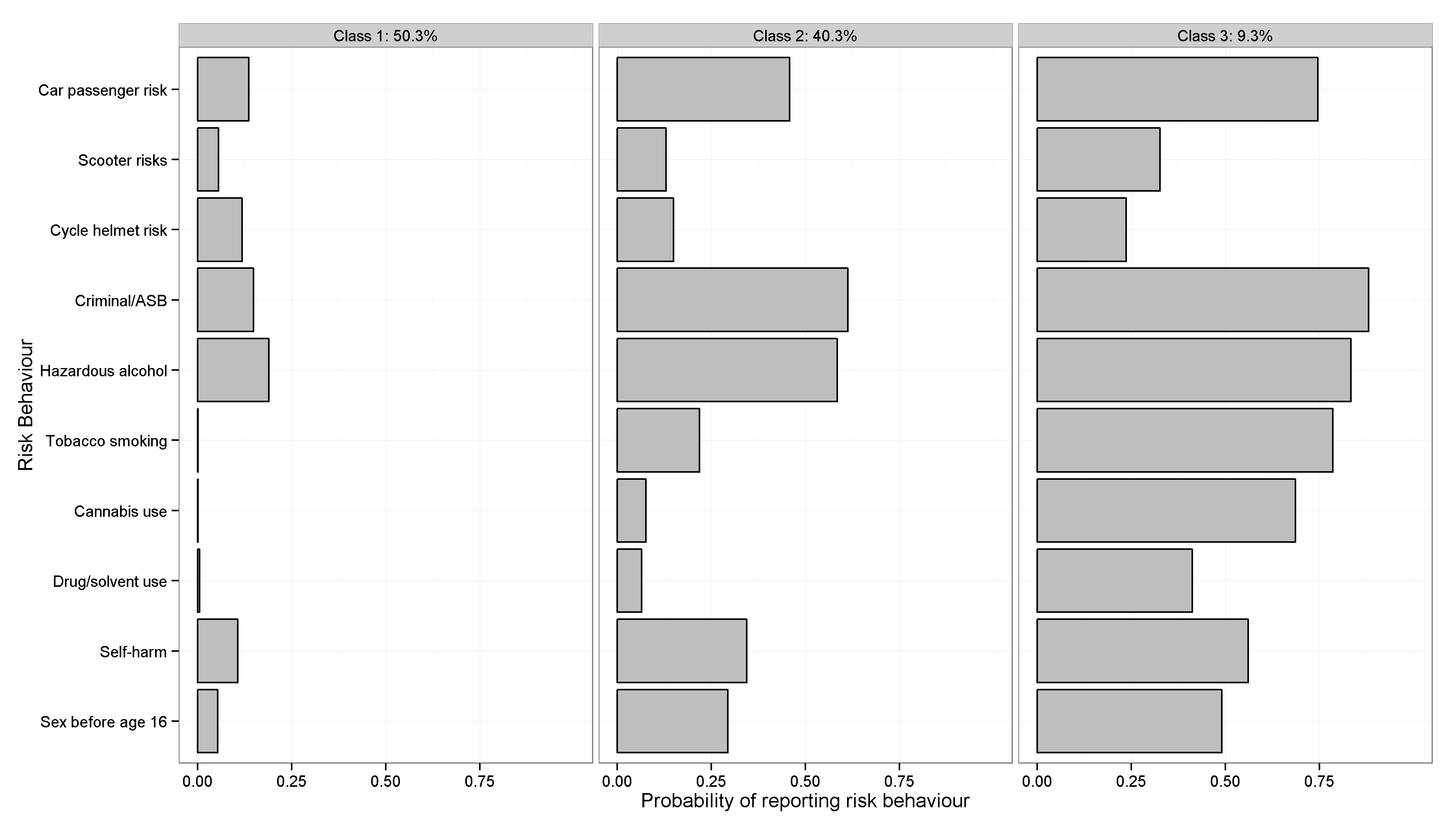


**Supplementary Figure 4. Bivariate residuals for 3 and 4-class males’ models (complete case sample (S1))**


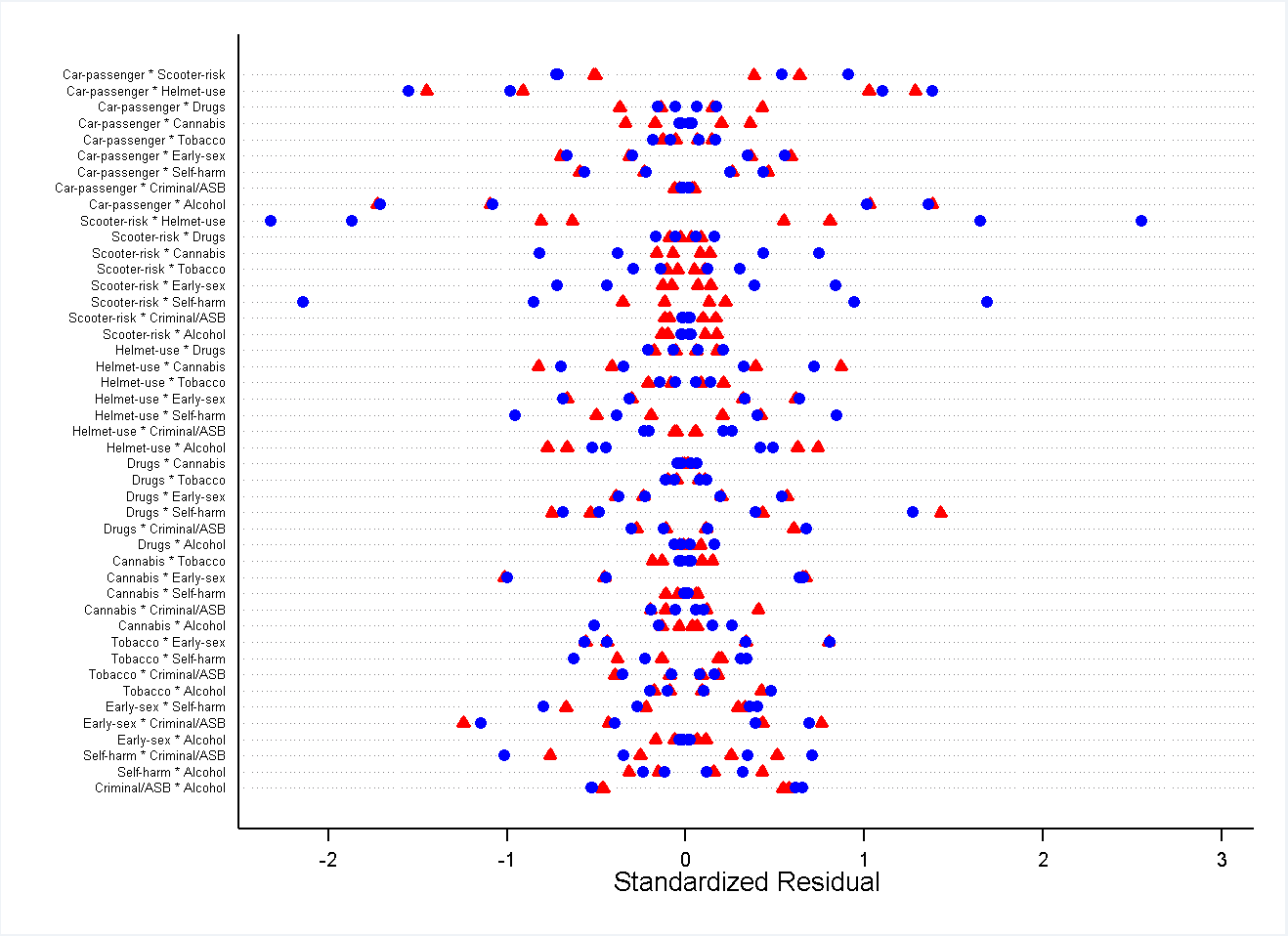


**Supplementary Figure 5. Bivariate residuals for 3 and 4-class females’ models (complete case sample (S1))**


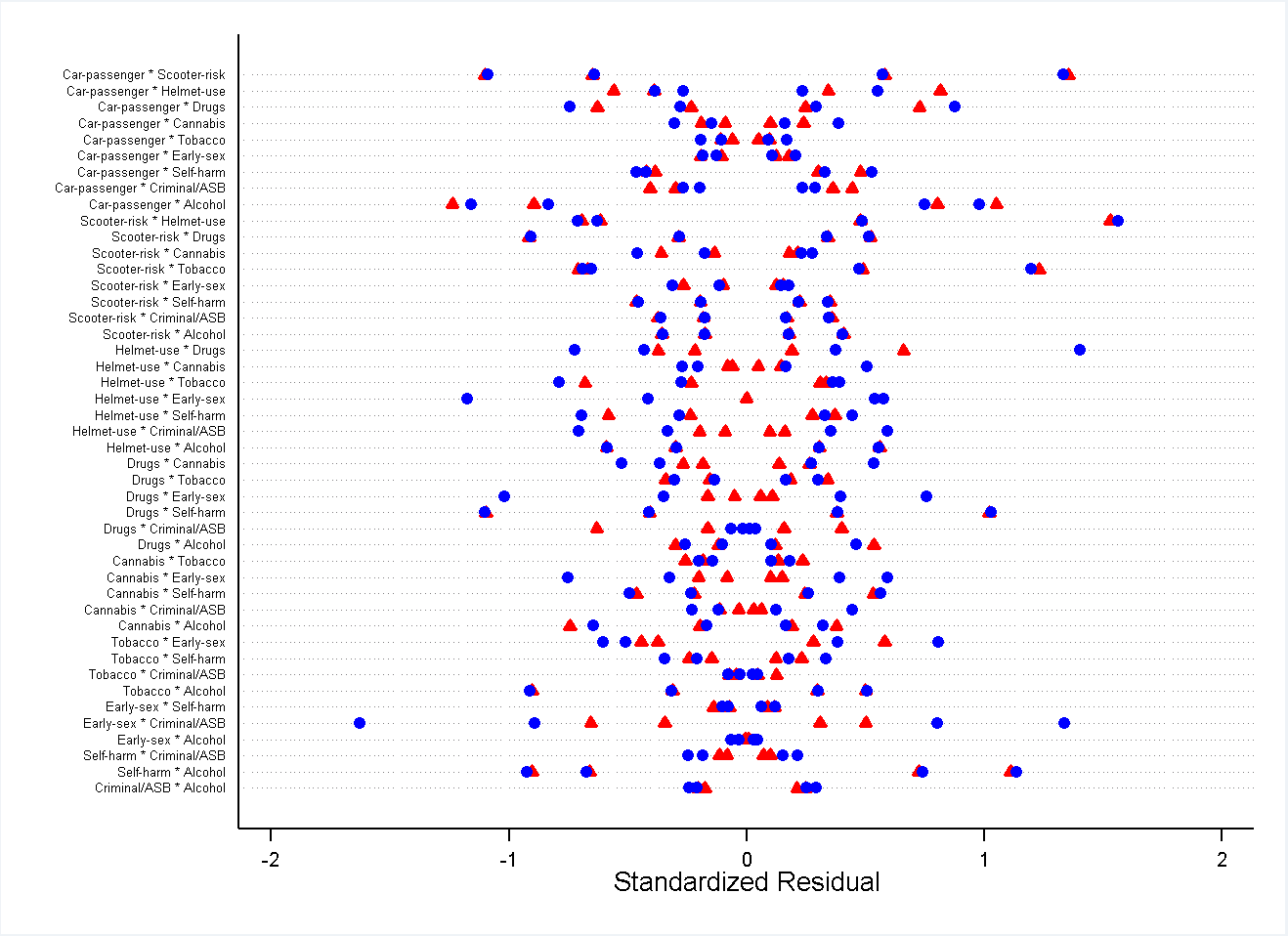


Blue circles = 3-class model, red triangles = 4-class model.

1. Melotti R, Heron J, Hickman M, Macleod J, Araya R, Lewis G. Adolescent alcohol and tobacco use and early socioeconomic position: The ALSPAC birth cohort. Pediatrics. 2011;127:e948-e55.

2. Gregg P, Propper C, Washbrook E. *Understanding the Relationship between Parental Income and Multiple Child Outcomes: a decomposition analysis*. Bristol, UK: 2008 2008. Report No.: 08/193.

3. Cole TJ, Freeman JV, Preece MA. Body mass index reference curves for the UK, 1990. Archives of Disease in Childhood. 1995;73(1):25-9.

4. Lewis G, Pelosi AJ, Araya R, Dunn G. Measuring psychiatric disorder in the community: a standardized assessment for use by lay interviewers. Psychological medicine. 1992;22(2):465-86.

5. Bell T, Watson M, Sharp D, Lyons I, Lewis G. Factors associated with being a false positive on the General Health Questionnaire. Social psychiatry and psychiatric epidemiology. 2005;40(5):402-7.

6. Saunders JB, Aasland OG, Babor TF, de la Fuente JR, Grant M. Development of the Alcohol Use Disorders Identification Test (AUDIT): WHO Collaborative Project on Early Detection of Persons with Harmful Alcohol Consumption--II. Addiction. 1993;88(6):791-804.

7. Holtgraves T. Evaluating the problem gambling severity index. Journal of gambling studies / co-sponsored by the National Council on Problem Gambling and Institute for the Study of Gambling and Commercial Gaming. 2009;25(1):105-20.

8. Ferris J WH. The Canadian Problem Gambling Index. Canadian Centre on Substance Abuse, 2001.
